# Innovative Fiber-Reinforced Polymer Rope-Based Closed-Form Retrofitting Methods Applied in Reinforced Concrete T-Shaped Beams under Torsion

**DOI:** 10.3390/polym16182634

**Published:** 2024-09-18

**Authors:** Adamantis G. Zapris, Violetta K. Kytinou, Constantin E. Chalioris

**Affiliations:** Laboratory of Reinforced Concrete and Seismic Design of Structures, Civil Engineering Department, School of Engineering, Democritus University of Thrace, 67100 Xanthi, Greece; azapris@civil.duth.gr (A.G.Z.); chaliori@civil.duth.gr (C.E.C.)

**Keywords:** torsion, reinforced concrete (RC), T-beams, strengthening, fiber reinforced polymer (FRP) strips, FRP bundles, FRP ropes, U-shaped jacketing, FRP debonding, closed-form retrofitting

## Abstract

The fiber-reinforced polymer (FRP) strengthening of reinforced concrete (RC) elements with torsional deficiencies has not yet been extensively studied. Existing studies have primarily focused on rectangular RC beams. The few studies on L or T-shaped beams have used open-form retrofitting methods. However, premature debonding of the retrofitting from concrete surfaces often leads to detachment before achieving enhanced torsional capacity. This study introduces an innovative application of closed-form FRP retrofitting for RC T-beams against torsion. Two novel closed-form torsional upgrading methods were proposed and investigated through a comprehensive experimental program involving eight large-scale T-beams. One method employs FRP ropes embedded in transverse grooves near the surface, while the other combines U-shaped EB-FRP strips with FRP ropes. Additionally, two configurations were examined replicating scenarios where the upper part of the slab is accessible or inaccessible. The results demonstrate that the closed-form methods improve torsional strength by 9% to 25% and twist at failure by 92% to 536% compared to unstrengthened beams, with beams retrofitting through the slab exhibiting superior performance. Step-by-step technical guidelines of the proposed methods are presented to minimize construction defects and ensure effective implementation in real RC structures.

## 1. Introduction

Existing reinforced concrete (RC) structures may require different retrofitting to rehabilitate or upgrade their capacity and overall performance for various reasons, such as reinforcement deficiencies [1,2], damages [3,4], changes in building use, increased live loads, and modifications of the Standard Code requirements. Over the past few decades, there has been a significant focus on developing effective retrofitting techniques to address these challenges [5,6,7]. Among the various methods studied, fiber-reinforced polymer (FRP) composites have gained popularity due to their ease of application [8,9,10,11,12] and their ability to enhance strength and ductility without significantly altering the dimensions and the dynamic properties of the retrofitted RC elements [13,14,15,16,17]. The advantages of FRP materials vary based on the type of fiber and resin used. Carbon FRPs (CFRPs), for example, are known for their high mechanical properties, as well as excellent fatigue, corrosion, and creep resistance [18]. In contrast, glass FRPs (GFRPs) and basalt FRPs (BFRPs) exhibit higher elongation at break and are more cost-effective, making them attractive options in certain applications [19]. Additionally, thermosetting resin composites are known for their good molding process and high mechanical properties, while thermoplastic resin composites offer recyclability, high toughness, and durability [20]. FRP materials have been thoroughly studied and successfully applied to address deficiencies and damages [21,22,23,24,25,26,27,28,29,30,31]. However, despite the extensive research [32,33,34,35,36,37,38], the study of FRP applications for enhancing the torsional resistance of RC elements has been comparatively limited [39].

Torsional damage is less commonly encountered in RC structures compared to other types of damages, such as shear and flexural, which has led to it being less frequently studied [40,41]. Nevertheless, when torsion occurs it undermines the shear capacity of RC beams and can result in brittle failure, which is undesirable, as it compromises the ductile behavior of the structure [42,43], highlighting the need for further study [44,45]. The complexity of torsional behavior, which is characterized by three-dimensional stress states and intricate crack patterns, has also contributed to the challenge of studying torsional effects [41,46,47]. Torsional stresses are more likely to occur in certain structural elements, such as spandrel beams, edge beams, L- and T-shaped beams, and beams subjected to eccentric or asymmetrical loading [48]. These elements are common in structures with irregular geometries, bridges, or those that incorporate architectural features like cantilevers, curved forms or overhangs. The importance of addressing torsional behavior in these elements cannot be overstated, as torsional failure can lead to severe structural deficiencies and, in extreme cases, even collapse [41].

The existing literature on torsional retrofitting of RC beams using FRP materials has primarily focused on retrofitting of rectangular cross-section beams [49,50,51,52,53,54,55,56,57]. Studies have explored both full wrapping configurations and open-form configurations, such as externally bonded (EB) U-shaped applications or side-bonded FRP materials applied to the sides of the beams [58,59,60,61,62]. Full wrapping has generally been found to be more effective in enhancing torsional capacity compared to open-form configurations [41,63]. However, this method has predominantly been applied to rectangular beams and does not address the complexities of T-shaped or L-shaped beams, where full wrapping is often impractical or impossible due to geometric constraints. The limited studies that have investigated torsional strengthening in T-shaped or L-shaped beams have applied open-form strengthening configurations [61,64,65]. While these techniques offer some improvement in torsional capacity, they are prone to premature debonding, which significantly reduces their effectiveness [61]. Studies have shown that when FRP debonding appears, the torsional resistance of the beam is compromised, leading to rapid progression of damage and eventual failure [41,66].

Furthermore, the few existing regulations and design guidelines [67,68,69] that provide specifications for torsional strengthening of RC elements with FRP materials primarily focus on EB full wrapping applications to rectangular cross-sections. While these guidelines emphasize the importance of closed-form configurations for effective torsional resistance, they do not address cases of non-rectangular cross-sections, such as T-shaped or L-shaped beams [70]. Consequently, applying these guidelines can be challenging is some cases, as in real structures, beams may often be surrounded by other elements, such as slabs, making full access for wrapping impossible.

This study aims to bridge this gap by introducing and investigating two innovative closed-form FRP rehabilitation methods for T-beams. The first method involves embedding FRP ropes in transverse grooves near the surface of the beam, while the second combines U-shaped EB-FRP strips with FRP ropes to form the closed-form configuration. Additionally, this study explores two different configurations for embedding the FRP ropes, one through the slab and the other through the web beneath the slab, to simulate scenarios where the upper part of the slab is either accessible or inaccessible. By doing so, the research addresses the practical challenges associated with strengthening T-beams in real structures. Through a comprehensive experimental investigation, involving eight large-scale T-beams, this study evaluates the effectiveness of the proposed methods in enhancing torsional resistance. By addressing the limitations of current techniques and aligning with existing guidelines, this study aims to offer effective practical solutions that can be readily adopted in retrofitting RC structures against torsion. 

## 2. Experimental Program

### 2.1. Description of Specimens

The experimental program consists of eight RC beams, each with a total length of 1.6 m. To replicate the presence of a slab and its monolithic connection to the beam in real structures, the middle section of each beam, which is 1050 mm long, is designed with a T-shaped cross-section. The beam’s web has a cross-section with a height of 300 mm and a width of 150 mm, while the slab has a height of 50 mm and a width of 300 mm. At both ends of the beam, extending 275 mm from either side, the cross-section transitions to a rectangular shape. This configuration facilitates the experimental setup, allowing for the placement of metal frames to establish the desired support conditions. The cross-sectional dimensions of these end sections are 300 mm in height and 150 mm in width.

**Figure 1:**
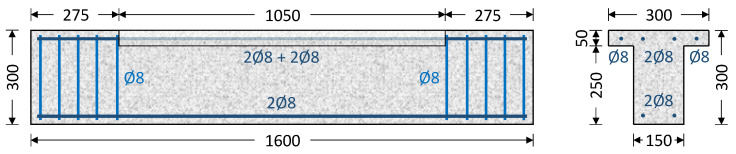
Geometric properties and specimen reinforcement arrangement in the section under study (dimensions in mm).

The longitudinal reinforcement was identical in all beams, consisting of four 8 mm diameter rods (4Ø8) that were positioned in the corners of the web cross-section. Furthermore, two 8 mm diameter bars (2Ø8) were installed in the slab area, with one bar per wing. The middle part of the beams, the one that was investigated, was left without transverse reinforcement in order to examine the impact of transverse FRP retrofitting on the torsional response. Dense 8 mm diameter transverse closed stirrups at a spacing of 50 mm were installed in the end sections of the beams. Furthermore, these end sections were strengthened with two layers of 0.331 mm thick carbon FRP sheet, providing high strength to prevent torsion-induced cracking and deformation in these areas. This setup ensures that any observed torsional damage will be concentrated in the middle section. Figure 1 depicts the geometric properties of the specimens and the configuration of the steel reinforcement.

The specimens were given characteristic designations to distinguish the way they were retrofitted with FRP materials in the section under investigation. Every designation begins with the letter “T”. In the case of retrofitting with EB-FRP strips (long and narrow pieces of sheets) around the perimeter of the beam’s web, the second designation is the letter “U”. The designation “U-F” denotes the closed-type retrofitting configuration utilizing an FRP bundle (rope) embedded through the slab, whereas the symbol “U-W” indicates that the closed-type configuration was implemented through the beam’s web. For example, the T-U-W notation describes a beam that was retrofitted with EB- FRP strips around the perimeter of the beam’s web and the closed-type retrofitting is achieved by inserting the FRP rope through holes in the web.

The letter “R” is included in the designation instead of “U” before “F” or “W” in cases where only FRP rope is used as a standalone torsional transverse reinforcement. The presence of “0.5” preceding “R” indicates the utilization of a cross-section that is half the size of a standard rope as specified by the manufacturer. Additionally, the number following “R” specifies the spacing at which the FRP material was installed. For example, the notation “T-0.5R75-F” describes a beam retrofitted with a rope, using a half-rope cross-section, installed at 75 mm distances, with the closed-type retrofitting performed through the slab.

The experimental program consists of eight beams. Out of the eight beams, one serves as a reference specimen (T-C specimen), while the remaining beams were retrofitted perpendicular to the longitudinal axis. Among these beams, one has an open type of FRP retrofitting (T-U), using EB-FRP strips around the perimeter of the beam’s web in a U-shaped configuration, also acting as a reference specimen. In the remaining retrofitted beams, closed-form retrofitting was applied using the two proposed methods. The combination of EB-FRP strips and FRP ropes was applied in two beams (T-U-F and T-U-W). The installation of FRP ropes placed in notches formed near the surface of the beams as standalone FRT torsional transverse reinforcement was applied in four beams (T-R150-F, T-R150-W, T-0.5R75-F, T-0.5R75-W). The geometric percentage of FRP torsional transverse reinforcement was maintained at a nearly constant level in all specimens to facilitate comparison, irrespective of the retrofitting method. Table 1 presents the reinforcement and FRP installation details for each specimen, including the geometric percentages of longitudinal steel reinforcement (*ρ_s,l_*) and FRP reinforcement (*ρ_f,v_*) calculated using the following equations.

(1) $$\rho_{s,l}=\frac{A_{s,l}}{A_{c}},$$

(2) $$\rho_{f,v}=\frac{A_{f}\cdot p_{f}}{A_{c}\cdot s_{f}},$$

In the equations provided, *A_c_* is the cross-sectional area of the concrete and *A_s,l_* is the total cross-sectional area of the longitudinal bars. *A_f_* is the area of the FRP material, either EB-FRP strip or FRP rope, and is calculated as *A_f_* = *n_f_* ·*t_f_* ·*w_f_* for strips, where *n_f_* is the number of layers of strips (*n_f_ =* 1 in this context), *t_f_* is the thickness of the sheet from which each layer of the strip is made, and *w_f_* is the width of the strip. For the rope, *A_f_* = *π* ·Ø*_R_*^2^ /4, where Ø*_R_* is the diameter of the rope cross-section. The perimeter of the FRP retrofitted cross-section can be calculated using two equations. The first equation, *p_f_* = 2·(*b_w_* + *h*), is used when the FRP wraps fully around the beam. The second equation, *p_f_* = *b_w_* + 2·(*h* − *h_f_*), is used when the FRP only retrofits the three sides of the beam. In both formulas, *b_w_* is the width of the beam’s web, *h* is the total height of the beam’s cross-section, and *h_f_* is the thickness of the slab. Finally, *s_f_* represents the spacing, along the beam axis, between two successive FRP positions, measured from their centerlines.

**Figure 2:**
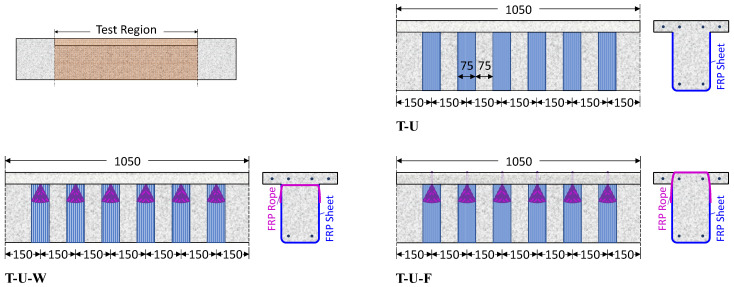
Arrangement of the EB-FRP strips: T-U specimen, T-U-W specimen with FRP rope through the web forming a closed-form retrofitting and T-U-F specimen with FRP rope through the slab forming a closed-form retrofitting (dimensions in mm).

The T-U beam was retrofitted with EB-FRP strips around the perimeter of the beam web, forming an open-type U-shaped retrofitting. The FRP strips were 0.331 mm thick, 75 mm wide, and placed with the fiber direction perpendicular to the longitudinal axis of the beam. Each strip had a net spacing of 75 mm from the next strip (Figure 2), resulting in a centerline distance of 150 mm between consecutive strips. The T-U-F and T-U-W beams were retrofitted with a combination of EB-FRP strips and FRP ropes, forming a closed-form configuration. The arrangement of the EB-FRP strips in these two specimens was the same as that in the T-U beam. In the T-U-W specimen, the FRP ropes were placed through holes in the web beneath the slab, replicating a scenario where the upper area of the slab is inaccessible (Figure 2). In the T-U-F specimen, the ropes were installed through holes and notches in the slab, simulating a situation where the upper area of the slab is accessible (Figure 2).

**Figure 3:**
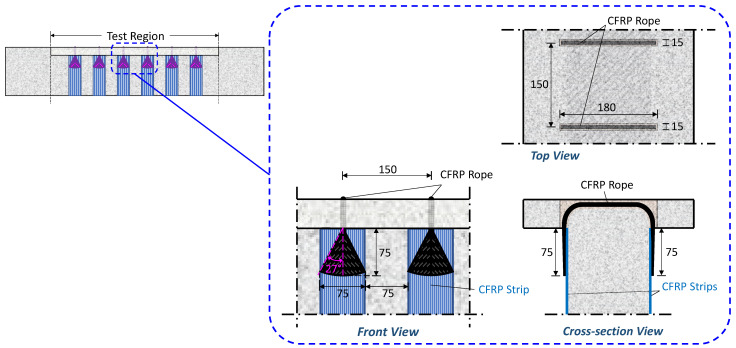
Detail of the closed retrofitting configuration with EB-FRP strips and FRP rope through the slab, T-U-F beam (dimensions in mm).

Each EB-FRP strip was combined with a rope to form the closed retrofitting configuration. The length of the rope was adjusted in each case to join the two ends of each strip, with its cross-section being slightly larger than the fiber content of the sheet, as recommended in the literature [71]. When forming the FRP application, the cross-section of the rope at the ends was gradually widened (formation of the fan) to attach it to the EB-FRP strips. The widening angle of the fan was 27°, with a length and width of 75 mm. Figure 3 provides a detailed representation of the configuration of the EB-FRP strip when the closed-form retrofitting is carried out through holes in the slab. A similar configuration was used when the retrofitting was carried out through holes in the web.

**Figure 4:**
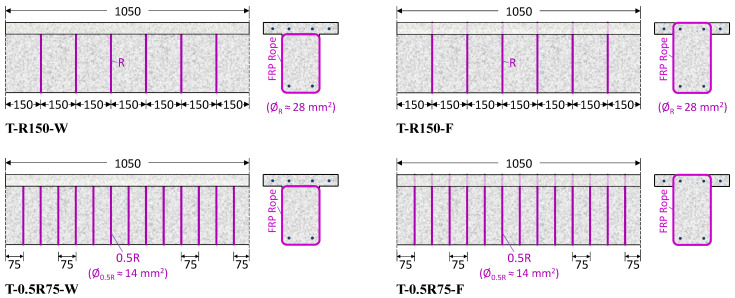
Arrangement of the FRP ropes placed in notches near the cross-section surface: Closed-form retrofitting configuration performed through the slab, T-R150-F and T-0.5R75-F beams, and closed-form retrofitting configuration performed through the web, T-R150-W and T-0.5R75-W beams.

The remaining specimens were retrofitted with unidirectional FRP bundles positioned perpendicular to the longitudinal axis of the beam in notches created near the beam surface. In the T-R150-F and T-0.5R75-F beams, the ropes were inserted in notches drilled around the perimeter of the beam and on the top surface of the slab, with the cross-section completely wrapped through holes perpendicular to the slab, representing the case of an accessible slab. On the other hand, the T-R150-W and T-0.5R75-W beams were completely wrapped with FRP ropes by drilling holes under the slab in the beam web, representing the case of an inaccessible slab. In the T-R150-F and T-R150-W beams, ropes of full cross-section were placed every 150 mm (Figure 4). In the T-0.5R75-F and T-0.5R75-W beams, half cross-section ropes were placed every 75 mm (Figure 4).

**Figure 5:**
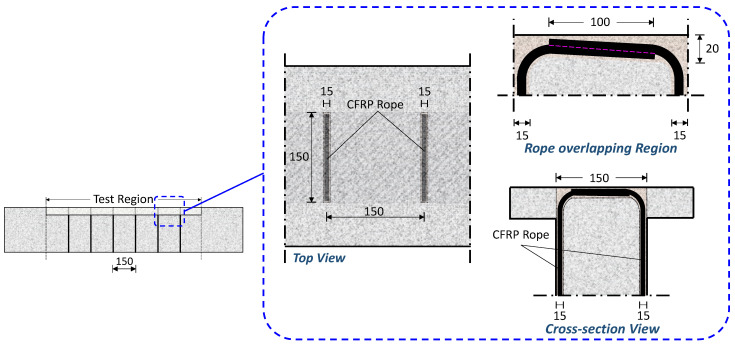
Detailed configuration of the external retrofitting using a closed-type stirrup by placing an FRP rope in notches near the cross-section surface through the slab, specimen T-R150-F (dimensions in mm).

In order to create a closed-type stirrup and avoid any slipping, the rope in the upper part of the slab (specimens T-R150-F and T-0.5R75-F) or inside the hole in the beam’s web (specimens T-R150-W and T-0.5R75-W) had an overlap length of about 100 mm. This length is equivalent to 10 times the diameter of the entire rope cross-section (Figure 5). The length of this overlap corresponds to the width of the beam following surface shaping procedures. Figure 5 depicts a comprehensive visual representation of the closed-type external retrofitting configuration using solely FRP rope. This closed-type FRP application is carried out by passing the rope through the slab. The same arrangement was utilized when the closed-form retrofitting was carried through holes in the beam’s web.

The notches drilled in the specimens had the same width and depth, measuring 15 mm, except for those in the upper part of the slab in the T-R150-F and T-0.5R75-F specimens, which had a slightly larger depth of 20 mm to accommodate the overlapping of the ropes. Similarly, the holes in the specimens, whether in the slab’s wings or in the beam’s web, were 16 mm in diameter. To prevent stress concentration and local fiber rupture, each corner at a strengthening placement point (such as the bottom edges of the web or the connection points of the notches or holes) was curved with a radius of curvature of 30 mm.

### 2.2. Materials

In order to assess the mechanical characteristics of the concrete during the casting of the beams, twelve cylindrical specimens with dimensions of Ø150 × 300 mm were also constructed. Six of these specimens were used to determine the compressive strength of the concrete, while the remaining six were used to determine the tensile strength through a tensile splitting test. The compression and tensile splitting tests were performed concurrently with the experimental testing of the beams. The mean compressive strength of the concrete was found to be 44.2 MPa, with a standard deviation of 1.9 MPa. The mean tensile strength was 3.1 MPa, with a standard deviation of 0.3 MPa. The steel reinforcement was classified as B500C. After conducting tensile testing on three steel bars, the average yield stress was determined to be 547 MPa, with a standard deviation of 1.45 MPa. The average ultimate tensile strength was 642 MPa, with a standard deviation of 7.48 MPa.

A unidirectional carbon fiber fabric (SikaWrap^®^-600C) was used for the construction of the EB-FRP strips. According to the manufacturer, after fiber impregnation, the nominal sheet thickness is 0.331 mm, the average tensile strength is 3.00 GPa, the average modulus of elasticity is 225 GPa, and the elongation at rupture is 1.33%. A two-component thixotropic epoxy resin (Sikadur^®^-300) was used to impregnate and adhere the sheet. The tensile strength of the resin is 45 MPa, its modulus of elasticity is 3.50 GPa, and its elongation at rupture is 1.5%.

Unidirectional carbon fiber FRP ropes (bundles) (SikaWrap^®^ FX-50C) were used to form a closed type of beam retrofitting, either in combination with EB-FRP strips or as the sole FRP material. The impregnated rope’s properties, as provided by the manufacturer, are as follows: the area of the rope is approximately 78 mm^2^ (with a dry fiber cross-section >28 mm^2^), the tensile strength is 2.00 GPa, the elastic modulus is 230 GPa, and the ultimate failure strain is 0.87%. Sikadur^®^-300 epoxy resin was used to impregnate the fibers, while Sikadur^®^-330 was used to embed and anchor the rope. The properties of the impregnation resin (Sikadur^®^-300) were reported in the preceding paragraph. Sikadur^®^-330 has a tensile strength of 30 MPa, a modulus of elasticity of 4.5 GPa, and an elongation at rupture of 0.9%. The main properties of the retrofitting materials are also summarized in Table 2.

### 2.3. Retrofitting Procedure

**Figure 6:**
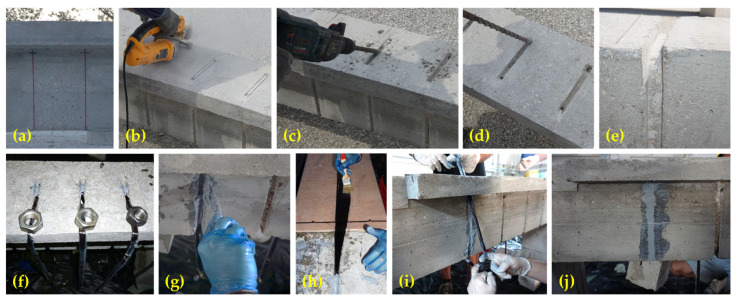
Step-by-step process of creating a closed-form stirrup using FRP rope in T-shaped RC beams (T-R150-W, T-R150-F, T-0.5R75-W, and T-0.5R75-F): (**a**) Marking retrofitting locations, (**b**) cutting notches, (**c**) drilling grooves, (**d**) drilling holes to connect grooves, (**e**) rounding edges, (**f**) impregnating and fixing a small portion of the rope end, (**g**) coating grooves with anchoring resin, (**h**) impregnating the remaining rope, (**i**) installing the rope, and (**j**) applying a small load for rope stretching and filling up grooves with resin.

A closed-form stirrup was created using an FRP rope near the surface in the beams T-R150-W, T-R150-F, T-0.5R75-W and T-0.5R75-F. The retrofitting locations were initially identified and marked (Figure 6a). Notches were cut using a circular saw (Figure 6b) and further refined into grooves by drilling with an impact drill (Figure 6c). In the slab section, and in the web section beneath the slab for specimens T-R150-W and T-0.5R75-W, holes were drilled to connect the notches (Figure 6d). The edges within the grooves and holes were rounded off (Figure 6e), and the grooves were thoroughly cleaned with compressed air.

The retrofitting was implemented in two stages to ensure proper installation of the ropes and to maximize the performance of the FRP material. Initially, a small portion of one end of each FRP rope was impregnated with resin and affixed to the upper part of the beam (Figure 6f). After placement, it was left undisturbed for a day to allow the resin to develop some initial strength. This small section acts as a fixed point to prevent the rope from slipping while pooling it at the end of the retrofitting process. Subsequently, the grooves were coated with anchoring resin (Figure 6g). The remaining part of the rope was then impregnated with resin (Figure 6h) and inserted into the grooves (Figure 6i). In order to achieve alignment and sufficient stretching of the fibers, a modest load was applied at the endpoint of the rope (Figure 6j). Finally, the grooves were filled with anchoring resin (Figure 6j).

Prior to the FRP application in T-U, T-U-W, and T-U-F beams, the positions were predefined and marked. An angle grinder was then used to remove the outer layer of concrete and round the edges of the web. For the T-U-F beam, additional holes were drilled perpendicular to the slab using an impact drill, and notches to accommodate the rope were created in the upper part of the beam with a circular saw. In the T-U-W beam, holes were drilled in the web, just below the slab, to facilitate the rope installation.

The FRP retrofitting application regions were meticulously cleaned using compressed air to remove any remaining dust and debris. The FRP strips were precisely cut to the required dimensions, impregnated with resin, and thereafter attached to the retrofitting region which was already coated with anchoring resin. A special roller was used to remove trapped air during the application. For beams with closed-type FRP configuration (T-U-F and T-U-W), after the strips were attached, the rope was also impregnated, and the notches and holes were coated with anchoring resin. The FRP rope was then inserted, and the fan was formed at both ends, ensuring proper length, opening angle, and consistent thickness.

### 2.4. Experimental Setup and Instrumentation

**Figure 7:**
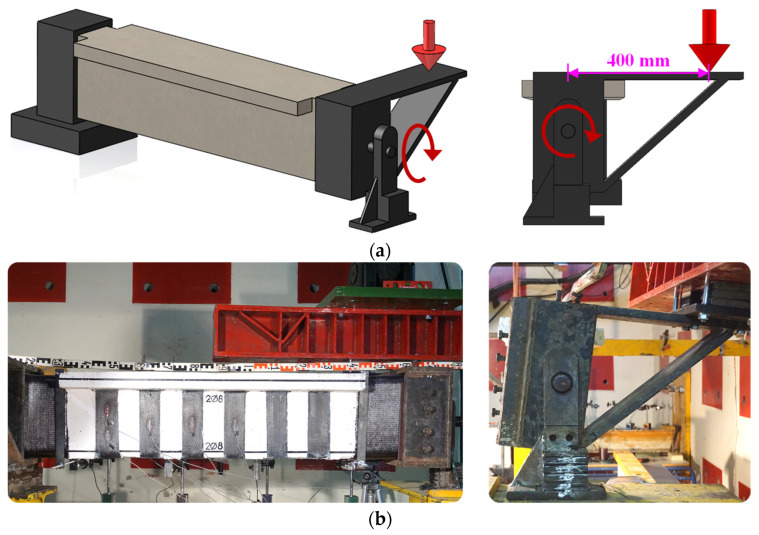
Experimental setup for torsional testing: (**a**) Schematic representation and (**b**) actual setup.

**Figure 8:**
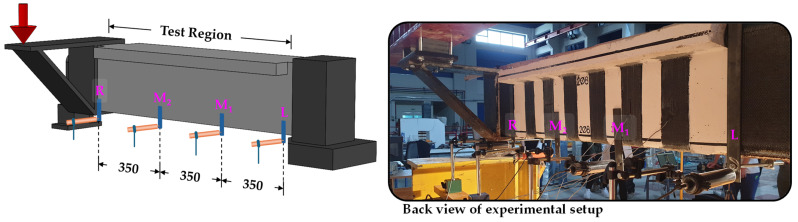
Locations of the LVDTs along the test region of the beam.

The experimental setup is shown in Figure 7. Figure 7a schematic representation and 7b actual setup). One end of the beams was fixed with a special metal assembly, while the other was free to rotate and deform along its longitudinal axis. A hydraulic actuator (MTS 201G2) was used to apply a torsional moment, which was transmitted through a 400-mm-long metal lever arm. The load was applied in displacement control mode at a constant rate of 0.02 mm/s and measured with a load cell. Linear variable displacement transducers (LVDTs) were placed horizontally at fixed distances from the beam centerline at the start, end, and 350 mm intervals along the section under investigation (4 LVDTs in total) (Figure 8). The LVDT measurements were used to determine the twist angle per unit length of the beam. Strain gauges were attached at characteristic points of the EB-FRP strips to measure strains, and crack width meters were used to track the progression of the main cracks.

## 3. Results

### 3.1. Visual Observation and Failure Modes

**Figure 9:**
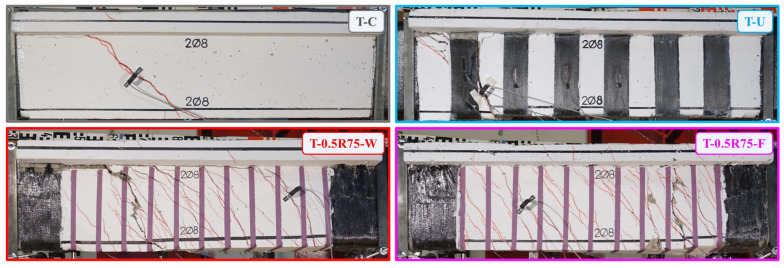
Typical failure modes of the specimens.

Figure 9 illustrates the final cracking patterns of the T-C, T-U, T-0.5R75-W, and T-0.5R75-F beams. These patterns represent the typical failure modes observed in the different retrofitting groups and the reference beam. All beams failed due to torsion, exhibiting helical shear cracks. The failure of the T-C and T-U beams was characterized by the development of a wide crack that ultimately led to beam failure. In the T-U beam, the failure was preceded by the premature debonding of the EB-FRP strip, caused by the propagation of the crack through the strip. This is a commonly observed failure of U-jacketed RC beams under torsion due to the failure at the concrete and the EB-FRP adhesive interface and several studies highlighted substantial reductions in the potential torsional capability [41,62].

**Figure 10:**
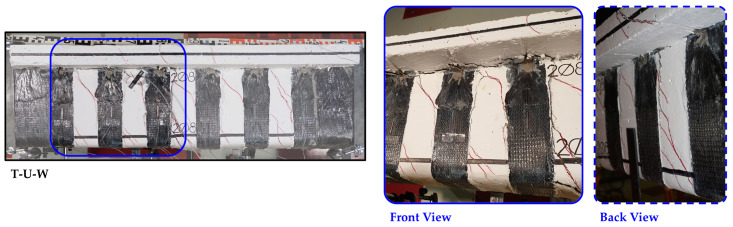
FRP rope anchors retaining U-shaped EB-FRP strips in place despite debonding from the substrate (T-U-W specimen failure).

In the beams with closed-form retrofitting configuration using a combination of U-shaped EB-FRP strips and ropes (T-U-W and T-U-F), multiple cracks formed before the final failure. Similar to the T-U beam, these beams also exhibited premature debonding of the U-shaped EB-FRP strips at low strain levels. However, the rope, with its fan-shaped formation at the ends, effectively anchored the EB-FRP strip, creating a closed-form configuration. This anchoring allowed the retrofitting to continue contributing to the beam’s response even after the debonding of the EB-FRP strip (Figure 10). Notably, no damage was observed at the connection point between the two segments (fan portion) of the closed-form retrofitting up to the point of final failure. It is noted that the recent work of Abdoli et al. [72] also demonstrated the effectiveness of FRP rope fan anchors to improve the post-peak torsional response, enhancing the ability of the EB-FRP retrofitted RC beam to induce significant torsional deformations.

The beams with closed-form retrofitting using only FRP ropes (T-R150-W, T-R150-F, T-0.5R75-W, and T-0.5R75-F) exhibited severe cracking upon failure. The occurrence of multiple cracks was more pronounced in the beams with a denser arrangement of FRP application (T-0.5R75-W and T-0.5R75-F) compared to those with sparser arrangements (T-R150-W and T-R150-F), despite maintaining the same geometric torsional transverse FRP percentage. In the beams where the closed-form configuration was applied through the beam’s web (T-R150-W and T-0.5R75-W), the critical crack was accompanied by horizontal cracking beneath the slab, which tended to separate the slab from the web. These observations are with accordance to the conclusions derived from the study of Abdoli et al. [73] that indicated the substantial impact of various factors of the torsional strengthening techniques, such as installation of FRP in grooves, adjusting the FRP spacing to width ratio, incorporating FRP anchoring, and extending FRP reinforcement to the FRP effective strain and to upgrade the performance of the strengthening system.

### 3.2. Main Experimental Response Values

Table 3 presents the values for the moment at the onset of cracking (*T_cr_*), the angle of twist per unit length at the onset of cracking (*θ_cr_*), the ultimate moment after cracking (*T_u_*), and the corresponding angle of twist per unit length (*θ_Tu_*). Additionally, the table includes the angle of twist per unit length at failure (*θ_0.8__Τ_*), defined as the point where the torsional moment drops at 80% of its ultimate value, which is considered a reliable measure of torsional strength. As expected, the torsional moments at the onset of shear cracking are consistent across the specimens, indicating minimal influence from the FRP retrofitting before the first diagonal crack forms. The negligible effect of the reinforcement on the pre-cracking response has also been evidenced by several experimental and theoretical studies [56,57,61]. The data show that the three FRP retrofitted beams exhibit an increase in torsional strength, ranging from 9% to 25%. The smallest increase, 9%, was observed in the T-U beam, which was retrofitted with U-shaped EB-FRP strips. This modest gain is attributed to the open-form configuration, where the strips quickly debonded. The greatest increase, 25%, occurred in the beam strengthened with a closed-form configuration through the slab, using densely spaced FRP ropes. Moreover, a significant increase was noted in the angle of twist per unit length at failure compared to the reference specimen, with increases ranging from 1.12 to 5.36 times. The results also indicate a notable distinction between the two closed-form FRP retrofitting configurations. The through-web configuration showed a maximum increase of 3.8 times, while the through-slab configuration exhibited an increase of 5.36 times compared to the reference specimen.

### 3.3. Torsional Moment Versus Twist per Unit Length Curves

**Figure 11:**
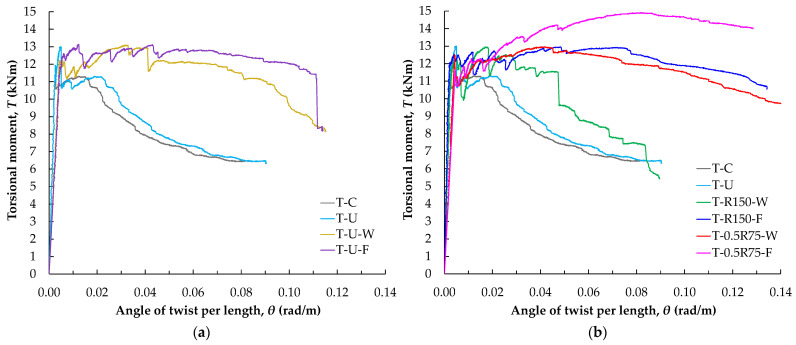
Comparative diagram of torsional moment versus twist per unit length for (**a**) T-C, T-U, T-U-W, and T-U-F beams, and (**b**) T-C, T-U, T-0.5R75-F, T-0.5R75-W, T-R150-F, and T-R150-W beams.

Figure 11a shows the torsional behavior of the beams, represented by the graph of torsional moment (*T*) versus the angle of twist per unit length (*θ*). The graph displays the response curves of different specimens: the reference specimen without retrofitting (T-C), the reference specimen with open-form retrofitting using U-shaped EB-FRP strips (T-U), and the specimens with closed-form FRP application using U-shaped EB-FRP strips and FRP ropes embedded either through the slab (T-U-F) or through the web (T-U-W).

The response of the beams, as depicted by the graph curves, provides valuable insights into the effectiveness of the proposed torsional retrofitting method. Initially, all beams exhibit elastic behavior with significant resistance to torsion. The torsional strength of the reference beam (T-C) decreases after concrete cracking, as it lacks both conventional and FRP reinforcement in the region under investigation. Specimen T-C exhibits the lowest torsional moment capacity, serving as a baseline for evaluating the effectiveness of the proposed retrofitting methods. The specimen with EB-FRP strips (T-U) shows that the FRP material as external torsional transverse reinforcement initially contributes to a modest increase in the torsional strength. However, this strength is not sustained due to premature debonding of the strips. Therefore, as it has recently been reported, it is essential to explore the efficiency of new anchorage systems and FRP configurations to concrete surface attaching to delay or eliminate the observed premature failure of the EB-FRP strips [40].

On the other hand, the beams retrofitted with closed-form FRP rope configurations (T-U-F and T-U-W) not only exhibit a modest increase in torsional capacity but also have the ability to endure substantially after cracking deformations prior to failure. These beams demonstrate a sustained level of strength across a wider range of post-elastic responses before encountering a reduction, suggesting that the use of closed-form reinforcement effectively delays the initiation of torsional failure mechanisms. The decline in torsional moment at higher deformations is more sudden for the T-U-W beam, suggesting that the closed-form retrofitting configuration through the beam web may be less effective in maintaining torsional capacity compared to the configuration through the slab (T-U-F).

Figure 11b depicts the torsional behavior of beams that have been retrofitted using closed-form FRP rope configurations, in comparison to the reference beam with U-shaped EB-FRP strips. The diagram displays the response curves of different specimens: the reference specimen with U-shaped EB-FRP strips (T-U), the specimen with only FRP rope placed in grooves every 150 mm and embedded through the web (T-R150-W), the corresponding specimen with the rope embedded through the slab (T-R150-F), the specimen with half rope cross-section placed at 75 mm intervals in grooves and embedded through the web (T-0.5R75-W), and the corresponding specimen with the rope embedded through the slab (T-0.5R75-F).

In all cases, the FRP retrofitted specimens demonstrate an increase in torsional moment capacity compared to the reference specimen, indicating that the FRP ropes, whether embedded through the web or through the slab, effectively enhance torsional resistance. Notably, beams with ropes embedded through the slab (-F) tend to perform better than those with ropes embedded through the web (-W), suggesting that the specific closed-form FRP configuration influences the torsional behavior. Beams retrofitted with half-rope cross-sections placed more closely together (at 75 mm intervals) exhibit even higher torsional resistance, as seen in specimens T-0.5R75-W and T-0.5R75-F. This indicates that, even with the same geometric FRP percentage, more densely spaced reinforcement can lead to greater torsional resistance. The denser and more uniform placement of the FRP material appears to contribute to a more evenly distributed resistance to torsion, which is crucial for preventing the widening of cracks and reducing the risk of failure. In the study of Askandar et al. [55], it has also been proved that increasing the FRP ratio using dense spacing between EB-FRP strips enhanced the ultimate torsional moment instead of using a higher number of FRP plies. The mechanism that influences the denser FRP reinforcement on load-bearing performance is attributed to the reduced spacing of transverse torsional reinforcement, which effectively restrains concrete and inhibits cracking development and propagation so that longitudinal bars can play a fuller role. Further, torsional stress is distributed on more FRP ropes, intersecting the diagonal cracks and reducing the strain on each FRP rope.

**Figure 12:**
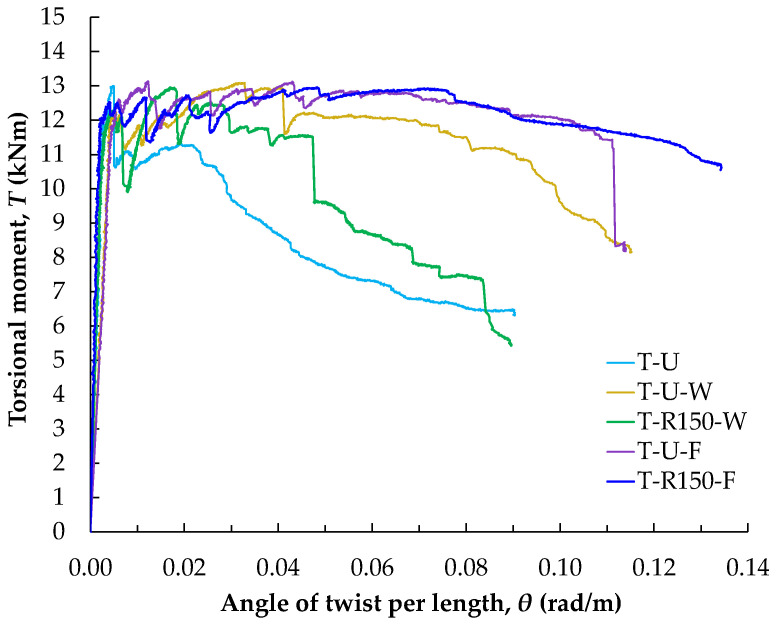
Comparative diagram of torsional moment versus twist per unit length for T-U, T-U-W, T-U-F, T-R150-W, and T-R150-F beams.

Figure 12 presents a comparison of the torsional response of beams retrofitted with the two proposed closed-form methods against the reference beam strengthened with U-shaped EB-FRP strips. The diagram specifically includes the response curves of the reference specimen with U-shaped EB-FRP strips (T-U) and the specimens with closed-form strengthening configured either through the slab (T-U-F and T-R150-F) or through the web (T-U-W and T-R150-W), arranged at 150 mm spacings. The results show that both proposed methods significantly improve torsional resistance compared to the reference beam (T-U), highlighting their effectiveness. The retrofitted beams with closed-form FRP configuration developed a continuous rectangular shear flow in the retrofitting system that prevented severe diagonal cracking. Therefore, the retrofitted beams exhibited high torsional deformability and improved overall response.

Among the FRP retrofitted specimens, those with ropes embedded through the slab (T-R150-F and T-U-F) display nearly identical responses, with similar increases in torsional strength and comparable post-cracking deformation. In these cases, the presence of FRP rope along the thickness of the flange section was the main reason that inhibited the development of cracking at the connection of the flange and web sections.

## 4. Design of the Connection Region of the Closed-Form Configuration

**Figure 13:**
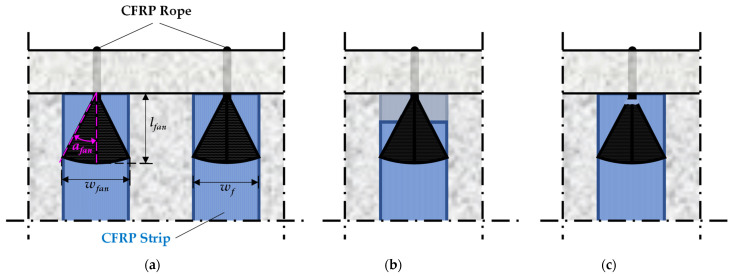
Connection area between an EB-FRP strip and FRP rope: (**a**) Geometry of the FRP fan, (**b**) debonding of the fan from the EB-FRP strip, and (**c**) fracture of the fan fibers.

The effectiveness of a closed-form configuration consisting of EB-FRP strips and FRP ropes depends greatly on the proper connection between the two segments. This connection is achieved by gradually widening the fibers of the rope to form a fan-shaped surface that is bonded to the EB-FRP strip. The fan should be carefully designed to avoid the possibility of failure. Proper fan design will allow the retrofitting to behave as a closed-form configuration, with failure characterized by fiber rupture. Conversely, failure of the fan will convert the configuration to an open form, which will fail primarily due to its debonding. Furthermore, since the fan is activated primarily after the EB-FRP strip has begun to debond from the surface of the structural element, the failure of the fan will often coincide with the simultaneous failure of the entire retrofitting configuration, which could result in a sudden and brittle failure. If the design ensures that failure is driven by the fiber fracture of the EB-FRP strips, the retrofitting could be simplified and approached with existing guidelines for torsional strengthening. The connection between the segments could fail if the fan debonds from the EB-FRP strip due to an insufficient bonding area (Figure 13b) or if the fan fibers fracture due to excessive tensile stress, exceeding the material’s tensile strength (Figure 13c).

The two modes of failure are primarily influenced by the configuration of the fan, making its design important for ensuring that it does not fail until the rupture of the EB strip fibers. This condition can be expressed in terms of the forces associated with each failure mode and their respective hierarchies. Specifically, the tensile force required to cause rupture of the FRP sheet fibers, *N_lr_*, should be less than the force required to cause fan debonding, *N_sd_*, and also less than the force needed to cause fan fibers, *N_fr_*.

(3a) $$N_{lr}\leq N_{sd},$$

(3b) $$N_{lr}\leq N_{fr},$$

The force leading to fan debonding can be calculated using the empirical Equation (4) [74], the force leading to fan fiber fracture can be determined using Equation (5) [75], and the force leading to sheet fiber fracture can be calculated using Equation (6).

(4) $$N_{sd}=0.122\cdot \sigma_{r}\cdot A_{fan},\;$$

(5) $$N_{fr}=3.6\cdot E_{a}\cdot \varepsilon_{afe}\cdot A_{d}^{0.56}\cdot \left(\frac{90{}^{\circ}-a_{fan}}{90{}^{\circ}}\right),\;\;$$

(6) $$N_{lr}=w_{f}\cdot t_{f}\cdot E_{f}\cdot \varepsilon_{fe}\;,\;$$

where *σ_r_* is the tensile strength of the resin, *A_fan_* is the area of the fan, *E_a_* is the elastic modulus of the rope used to form the fan, *ε_afe_* is the effective strain of the fan at failure, *A_d_* is the cross-sectional area of the rope, *a_fan_* is the angle of fiber widening to form the fan, *w_f_* and *t_f_* are, respectively, the width and thickness of the sheet to which the fan is bonded, *E_f_* is the modulus of elasticity of the sheet, and *ε_fe_* is the effective strain of the sheet at failure.

By inserting Equations (4) and (6) into Equation (3a) and solving for *A_fan_*, a lower limit for the required area of the fan is obtained to ensure that failure due to debonding does not occur. Given the area, the length, *l_fan_*, and the fan widening angle, *α_fan_*, can be determined using the full width of the FRP sheet or strip to which the fan is attached (Figure 13a). However, if the resulting angle is too large, the ability to transfer tensile force is reduced. Thus, for a given applied force, a greater percentage of fibers will be required and consequently a larger FRP rope area (Equation (5)). The smaller the widening angle, the more efficient the fan becomes, as more fibers align with the direction of the force being transferred. Therefore, the fan should be reshaped to achieve a relatively small widening angle. If achieving a smaller angle is not feasible, an alternative solution is to form multiple connection points, and multiple fans with smaller widening angles, rather than relying on a single fan. Finally, with the geometric characteristics of the fan established, inserting Equation (5) into Equation (3b) provides a lower limit for selecting the cross-sectional area, *A_d_*.

If the bonding area, specifically the fan, is designed according to the methodology presented above, then the EB-FRP strips will fail due to fiber fracture before any failure occurs within the fan. Consequently, the closed-form configuration with a combination of EB-FRP strips and FRP rope could be addressed in a simplified manner as strengthening with EB-FRP strips with a full wrap of the cross-section, without significant deviation of the results. Thus, following the above methodology, when complete wrapping of the cross-section with EB-FRP is not feasible, such as in beams connected to a slab, it is possible to design in a simplistic manner a closed form of strengthening in accordance with the torsional strengthening provisions of regulatory frameworks

## 5. Conclusions

In this study, two novel methods of closed-form torsional retrofitting for T-shaped RC beams were proposed and investigated. The first method employs FRP ropes placed in transverse notches near the surface of the structural element to create the closed-form FRP application. The second method combines EB-FRP strips with FRP ropes to achieve a closed-form retrofitting configuration. The experimental program comprised of eight beams, with two acting as reference specimens while the remaining six beams were retrofitted using the proposed closed-form methods. The study also examined two different configurations of the proposed closed-form configuration. The first configuration was employed by embedding the ropes through holes in the slab, and the other involved embedding the ropes through holes in the web beneath the slab, to replicate the scenarios where the upper side of the slab is either accessible or inaccessible. Besides the investigation of the effectiveness of the proposed methods, this study also provides step-by-step practical instructions for effectively implementing the proposed FRP retrofitting methods in the field. These instructions are intended to reduce construction defects and provide practical advice for applying the proposed methods in real RC structures. Based on the findings of this study, the subsequent conclusions are drawn.

- The use of closed-form FRP application against torsion appears to be a highly effective method with promising results. Beams with a closed-type configuration, either solely with FRP ropes or in combination with EB-FRP strips and FRP ropes, demonstrated similar torsional resistance mechanisms. These retrofitting methods led to an increase in torsional strength by 1.09 to 1.25 times and a significant improvement in twist at failure by 1.92 to 5.36 times compared to unstrengthened beam resulting in an overall improvement of the structural response.
- Compared to EB-FRP strips in a U-shape configuration around the perimeter of the web (open-form strengthening), both closed-form FRP retrofitting methods offer improved torsional response and effectively address the early debonding issues associated with conventional external bonding of strips. Specifically, the closed-form methods resulted in a 1.71 to 4.79 increase in twist at failure compared to the U-shape configuration.
- The proposed closed-form FRP application applied through the slab is considered more effective when comparing the two investigated configurations, as a greater part of the beam’s cross-section is engaged in the response. Specifically, the through-the-slab configuration demonstrated a 2.79-fold increase in twist at failure compared to the through-the-web configuration. However, if the application of FRP ropes through the slab is not feasible, the closed-type retrofitting configuration through holes in the web can also provide an excellent alternative offering a remarkable improvement in the overall response.
- The densification of FRP reinforcement while maintaining the same geometric percentage exhibits higher torsional strength (with a 1.25-fold increase compared to unstrengthened beams) and, most importantly, substantially improves post-elastic response compared to installing the same geometric percentage of the externally applied FRP torsional transverse reinforcement in a sparser arrangement.

The two proposed methods offer a simple, fast, and minimally invasive way to create a closed-form FRP strengthening. In most cases where the sides of a beam are accessible, this approach is feasible and unaffected by the monolithic beam-to-slab connection. However, when one face of the beam is inaccessible, such as with an end beam adjacent to a neighboring building, creating a closed strengthening system becomes more challenging. When using only FRP rope as retrofitting, the proposed closed-form configuration is even easier and faster to apply as there is no need to design and shape the fan as in the case of a combination of strips with FRP ropes.

## Figures and Tables

**Table 1:** Steel and FRP reinforcement characteristics of the beams.

Specimen	Reinforcement (mm)	FRP Retrofitting				Reinforcement Geometric Percentage (%)	
	Longitudinal	Type ^1^	Closed-Form Configuration ^2^	*A_f_*	*s_f_*	Longitudinal Steel Bars	Transverse FRP Materials
				(mm^2^)	(mm)	*ρ_s,l_*	*ρ_f,v_*
T-C	4Ø8 + 2Ø8	―	―	―	―	0.574	―
T-U		ΕΒ	―	25	150		0.205
T-U-W		ΕΒ	ETW	25	150		0.252
T-U-F		ΕΒ	ETF	25	150		0.284
T-R150-W		Ropes	ETW	28	150		0.255
T-R150-F		Ropes	ETF	28	150		0.292
T-0.5R75-W		Ropes	ETW	14	75		0.255
T-0.5R75-F		Ropes	ETF	14	75		0.292

^1^ Type of FRP material: EB: Externally bonded FRP strips (long and narrow pieces of sheets) placed around the perimeter of the web, with a nominal thickness of 0.331 mm and a width of 75 mm. Rope: FRP bundle (rope) placed in grooves near the surface, with a dry fiber cross-section exceeding 28 mm^2^, as per the manufacturer’s specifications. ^2^ Closed-form configuration: ETW: Closed-form retrofitting with FRP rope embedded through holes in the web cross-section. ETF: Closed-form retrofitting with FRP rope embedded through holes in the slab and grooves in the upper part of the slab.

**Table 2:** Properties of FRP retrofitting materials.

Properties	SikaWrap^®^-600C	SikaWrap^®^ FX-50C	Sikadur^®^-300	Sikadur^®^-330
Modulus of elasticity (GPa)	225	230	3.5	4.5
Ultimate strain capacity (%)	1.33	0.87	1.5	0.9
Ultimate tensile strength (MPa)	3000	2000	45	30

**Table 3:** Main experimental results of the specimens.

Specimen	*T_cr_*	*θ_cr_*	*T_u_*	*θ_Tu_*	*θ_0.8T_*	*T_u_* Increase Ratio	*θ_0.8T_* Increase Ratio
	(kNm)	(rad/m)	(kNm)	(rad/m)	(rad/m)		
T-C	11.94	2.6 × 10^−3^	-	-	25 × 10^−3^	-	-
T-U	11.94	2.6 × 10^−3^	13.00	4.8 × 10^−3^	28 × 10^−3^	1.089	1.12
T-U-W	12.21	4.0 × 10^−3^	13.09	31.6 × 10^−3^	95 × 10^−3^	1.096	3.80
T-U-F	12.61	6.0 × 10^−3^	13.13	12.3 × 10^−3^	112 × 10^−3^	1.100	4.48
T-R150-W	12.28	4.8 × 10^−3^	12.96	17.0 × 10^−3^	48 × 10^−3^	1.085	1.92
T-R150-F	12.08	2.4 × 10^−3^	12.95	48.1 × 10^−3^	134 × 10^−3^	1.085	5.36
T-0.5R75-W	12.50	4.0 × 10^−3^	12.96	40.2 × 10^−3^	126 × 10^−3^	1.085	5.04
T-0.5R75-F	11.64	4.0 × 10^−3^	14.92	14.9 × 10^−3^	129 × 10^−3^	1.250	5.16

## Data Availability

The data supporting the findings of this study are available from the corresponding author upon reasonable request.
